# Social and Psychological Predictors of Youths’ Attitudes to Cryptocurrency

**DOI:** 10.3390/bs9120118

**Published:** 2019-11-20

**Authors:** Maria Gagarina, Timofey Nestik, Tatiana Drobysheva

**Affiliations:** 1 Department of HR Management and Psychology, FSEE HE Financial University, Moscow 125993, Russia; 2 Laboratory of Social and Economic Psychology, FSFES Institute of Psychology, Russian Academy of Sciences, Moscow 129366, Russia; nestik@gmail.com (T.N.); tdrobysheva@mail.ru (T.D.)

**Keywords:** globalization, attitude, cryptocurrency, Bitcoin, new technologies, trust, value orientations, moral foundations, economic socialization

## Abstract

The objectives of the study were to verify the “Attitudes Toward Cryptocurrencies Questionnaire” and to identify predictors of attitudes toward Bitcoin. Sample: 262 participants aged 17 to 30, of which 45% were male. Methods: Associations, “Value Scale”, “The Moral Foundations Questionnaire”, “Money Beliefs and Behaviors Scale” by A. Furnham, and “The Baseline Confidence Scale” by L. Haff. Confirmatory factor analysis proved the three-factor structure of the questionnaire. A linear regression analysis showed that beliefs in the potential of cryptocurrency as a payment instrument are directly related to people-centered care and value of freedom, and are inversely related to sanctity; they are associated positively with confidence in the financial system and negatively with confidence in the government. Age and gender also matter. Worries about the introduction of cryptocurrency are directly related to a negative attitude to money, the value of self-confidence, age, and confidence in the financial system and government, and are inversely related to trust in people and money anxiety. Willingness to use cryptocurrency in perspective directly depends on trust in the power of money, orientation towards independence in actions, age, and gender, and is inversely dependent on confidence in the government. The data state that the intention to use cryptocurrency is directly related to the desire for financial autonomy and distrust of social institutions.

## 1. Introduction

Experts explain the emergence of cryptocurrency in Russia through the processes of globalization, the leading feature of which is “the emergence of a multipolar world system and the growth of cultural diversity” [1] (p. 58), [2,3]. The effects of the globalization processes are manifested in various aspects: Changes in the peoples’ traditional ways of life, transformation of their worldviews, and shifts in cultural values behavioral stereotypes. As a result, the problem of preserving the value system specific to the Russian mentality has been exacerbated. The analysis of the effects of globalization processes most often has a negative modality. However, the study of ideas, opinions, judgments, and attitudes towards new economic phenomena—considered as the effects of globalization processes in our society—also presupposes a positive direction by finding mechanisms for the adaptation to a changing society.

The study of the attitudes of representatives of different groups of the population to new forms of money is connected with the analysis of the content and processes of the secondary and transitional forms of economic socialization (ES) [4,5]. Successful ES of young people results in the formation of socio-economic competence, which is understood as the ability to use their resources (knowledge, skills, experience) for the independent solution of life tasks related to their financial and material self-sufficiency and provision for their relatives. In this regard, the willingness of young people to use new forms of money (payment instruments) and their attitude to cryptocurrency in general can be considered as a criterion for their successful ES.

The difficulties of solving the problem posed in the work were due to a number of circumstances. The first is connected with the analysis of the connotations of “cryptocurrency” in the social representations of young people—whether cryptocurrency is perceived as money or treated in some other way; for example, as a new technology. The second circumstance was determined by the lack of psychological methods (in Russian) that can effectively solve the tasks posed in the work. Finally, existing studies of cryptocurrency are often sociological in nature or built in the context of an economic and legal approach with an appropriate disciplinary analysis [6,7]. That is, at present, many issues of the socio-psychological determination of attitude towards a new economic phenomenon are not reflected in the work of psychologists, except for in individual articles [8]. This study will make it possible to not only analyze the psychological nature of the phenomenon itself, but also reveal the mechanisms of economic and psychological adaptation of young people to the financial and economic effects of global processes in Russia.

The debate on the status of cryptocurrency is currently continuing both on the pages of scientific literature and in the media and public discourse [9]. However, it remains an open question whether cryptocurrency should be considered money and whether it perform all of the functions of money. The latter is due to the fact that only those tools that can perform functions dictated by the market can be retained as money in the economy [10]. We consider the following functions: Unit of account, medium of circulation, means of payment, means of accumulation and savings, and world money. According to experts, Bitcoin as a currency does not satisfy the two main criteria for money— multi-functionality and stability [11]; its characteristics do not correlate with the characteristics of traditional assets such as stocks, bonds, and commodities, both in stable times and in periods of financial turmoil [12]. It is noted that Bitcoin does not perform the function of a medium of exchange; it is more often used as a speculative investment. However, according to S. Ammous [13], Bitcoin is the only cryptocurrency that has the potential of a store of value because of the relative reliability and predictability of the offer (compared to other cryptocurrencies). This circumstance served as the basis for choosing Bitcoin (and not another type of cryptocurrency) as the subject of our study.

Unlike financial experts who interpret the phenomenon of cryptocurrency from the standpoint of its functionality and limitations, this phenomenon is perceived in public discourse as a new form of electronic money that allows one to quickly improve their financial status [14]. In this regard, for psychologists, it is important to identify and follow the analysis of ideas and attitudes to cryptocurrency—in particular, Bitcoin.

According to P. Webley and St. Lea, the coexistence of several forms of money (cash, bank accounts, credit and debit cards, gift tokens, etc.) in modern society indicates that people have different types of financial needs [15]. As research shows, different forms of money evoke different feelings and are spent in different ways. For example, in general, Russians are characterized by a low level of trust in monetary innovations, a predominance of the desire to have cash, and a lack of trust in other money forms [16]. In a study of See-To et al., 2018 [17] differences in consumer behavior in situations with three alternative payment technologies (cash, credit cards, and contactless smart cards) were found: The preference for a certain source of expenses is determined by consumers’ perception of the security of payment. Returning to the problem of identifying the attitude of Russians to cryptocurrency as a payment instrument, we note that its innovative nature and high level of technology are factors that complicate the process of perception and understanding of the phenomenon of “cryptocurrency”, which in turn can cause phobias [18].

When choosing the main construct—the category of "attitude"—we relied on the theory of Russian psychologist V.N. Myasishchev [19], according to which the relation includes three components (cognitive, emotional, and behavioral), as well as the approach to the study of the “psychological relations” of V.P. Poznyakov, who supplemented the three-component structure of the phenomenon with a fourth: The value component [20].

Studies of the phenomenon of "attitude to money" in Russian economic psychology have been most actively conducted since the beginning of the 1990s in connection with the socio-economic transformations in society that took place at that time. Most of the authors in their research models included an analysis of individual psychological, personal, socio-psychological, and social factors that determine the intensity of their attitude to money according to different scales laid down in the methodology. Thus, the relationship between social and money attitudes [21,22] and the economic activity of an individual [23] is shown, as well as the dependence of a relation on personal maturity [24], connection with social capital [25], religiosity [26], and ethical categories [27]. The value aspects of the attitude to money are touched upon by a number of authors in the context of the analysis of the function of value orientations as predictors of attitude [28]. Another aspect of the study of the phenomenon under consideration is related to the functions of the attitude towards money in different situations, like economic and psychological adaptation to changing conditions of life. In particular, the severity of financial anxiety (as a component of attitude to money) is considered as one of the indicators of a person’s subjective economic well-being [29], which performs the compensatory function of depriving psychological sovereignty [30], reduces the experience of the fear of death [31], and helps to cope with the situation of unemployment [4].

Based on the above analysis, the models of our research will consider the following characteristics as social and psychological factors: Value orientations, moral foundations, the level of confidence, and attitude to money.

Therefore, the purpose of the study was the identification and subsequent analysis of the attitude of young people to cryptocurrency and the factors causing it.

Due to the lack of development of the construct at the first stage of work, we planned to analyze the connotations of the concept of how respondents relate to cryptocurrency: As money, as an economic phenomenon, or as a modern technology. The second stage was intended to identify the most significant elements of the relationship to cryptocurrency and to analyze their manifestation and connection with socio-psychological factors.

The hypothesis of the study included the assumption that the attitude towards cryptocurrency as a new socio-economic phenomenon will be associated with the confidence in state and other social institutions, with the attitude toward money and earnings, as well as with such socio-psychological factors as moral foundations and value orientations.

The hypothesis of the study is specified in the tasks. Thus, at the empirical level, the nature of respondents’ attitudes to cryptocurrency, the representations of the components of the phenomenon under study, as well as the totality of socio-psychological factors associated with each of the components will be analyzed. The obtained results will allow us to determine the significance of the studied phenomenon in the process of economic socialization of young people in the context of the globalization in modern Russian society.

## 2. Materials and Methods

The methodological task was associated with the design and verification of the author’s “Attitudes Toward Cryptocurrency Questionnaire” for the subsequent execution of the main phase of the study. The development of the questionnaire included several stages: Formulating statements in accordance with the model of the phenomenon, checking reliability, eliminating unreliable items, and confirmatory analyses.

The sample: The study was attended by representatives of groups of young people—Bachelor and Master students, as well as those getting the second higher education from different universities in Moscow: Moscow State University (Psychology)—20%, Financial University (HR Management)—30%, State Academic University for Humanities (Psychology)—20%, Moscow Medical University REAVIZ (Faculty of Dentistry)—20%, not identified—10%. N = 263; 45% male, 55% female; aged from 17 to 30 years old (M = 23.3 years, SD = 4.5); 17–20 years 50%; 21–25 years 26%; 26–30 years 24%. Student participation in the research was voluntary; they were informed about the purpose of the research. There were no monetary benefits for participating, but participants were told that information obtained in the research could improve understanding of psychological aspects of cryptocurrency operations. Researchers paid special attention to the confidentiality in data collection, processing, and storage.

The choice of the group of respondents was based on the following: A group of students was taken, as we studied the attitude to cryptocurrencies in the context of primary economic socialization; the choice of non-economic disciplines was made to avoid the influence of education on attitude. According to studies of Bohr et al. in 2014 [32], the average age of Bitcoin users is about 30 years, but the aim of our research was to study not the behavior (mining, use of Bitcoin), but representations and attitude to the phenomenon among youths, not among specialists.

The preliminary stage of the research (pre-study) included solving the problem of identifying the semantic field of the concept of “Bitcoin”. For this purpose, an associative test was used. 

At the main stage of the study, the authors’ “Attitudes Toward Cryptocurrencies Questionnaire” (T.A. Nestik, T.V. Drobysheva) was developed. 

To research the socio-psychological factors related to attitude to Bitcoin, the following set of methods were included: The “Value Scale” of E.B. Fantalova to measure value orientations, and the “Moral Foundations Questionnaire (MFQ)” of J. Haidt (adapted for Russian samples by O.A. Sychev). This questionnaire suggests the following understanding of morality: Morality is a system of methods and criteria for evaluating actions as right and wrong. The area of morality can be streamlined by highlighting the following moral foundations: Care/harm, fairness/cheating, loyalty/betrayal, authority/subversion, and sanctity/degradation. To measure the attitude of the individual to money, we used a short version of the “Money Beliefs and Behaviors Scale (MBBS)” of A. Furnham (adapted for Russian samples by O.S. Deineka); to measure the level of confidence, we used “The Trust Scale” of L. Haff and L. Kelly (adapted for Russian samples by T.A. Nestik). Socio-demographic characteristics of the respondents were studied using questionnaires.

For statistics calculation, we used SPSS 23.0: Frequency analysis, confirmatory factor analysis, and regression analysis.

## 3. Results

### 3.1. Verification of the Questionnaire "Attitudes Toward Cryptocurrencies Questionnaire”

The theoretical model of the attitude to cryptocurrency included four components: Belief in the potential of cryptocurrency as a payment instrument (cognitive), emotional experiences about the introduction of cryptocurrency (affective), willingness to use cryptocurrency in perspective (behavioral), and the importance of cryptocurrency (value).

The first version of our questionnaire consisted of 22 statements grouped into scales according to the four-component structure of the attitude: Cognitive (for example, “The development of cryptocurrency is as inevitable as scientific and technical progress.”), affective (for example, “Cryptocurrency opens up unlimited possibilities for financial fraud.”), behavioral (for example, “I would buy cryptocurrency if I had enough resources.”) and value (for example, “For me, the value of cryptocurrency means that it can solve many of my problems in the future."). We calculated Cronbach’s alpha as a measure of internal consistency of each scale. Scales for cognitive, affective, and behavioral components of attitude showed high reliability (internal consistency; Cronbach’s alpha > 0.7). The questions of the value component of the attitude were not included in the final version of the questionnaire due to the low consistency of the scale (Cronbach’s alpha = 0.215). 

Models with single-factor, two-factor, and three-factor solutions were compared (Table 1).

Table 1 reveals the Chi-square value and minimum value of the discrepancy function (CMIN) for all models. For the three factor model, CMIN = 0.750 for the degree of freedom (df) = 26 and *p* = 0.814 (*p* > 0.05); the CMIN value is insignificant, and it indicates that the data fit the model. Table 1 also reveals RMR, GFI, and CFI (root mean square residual, goodness of fit index, and comparative fit index). For the three-factor model, the RMR = 0.048; this is less than 0.05, which suggests that the data fit the model. The GFI = 0.986 and CFI = 1.00, which are above the threshold value of 0.9 and also confirm the model. For the three-factor model, the root mean square error of approximation (RMSEA) = 0.995; this is less than 0.05, and also indicates that the data fit the model of attitude toward cryptocurrency.

**Figure 1:**
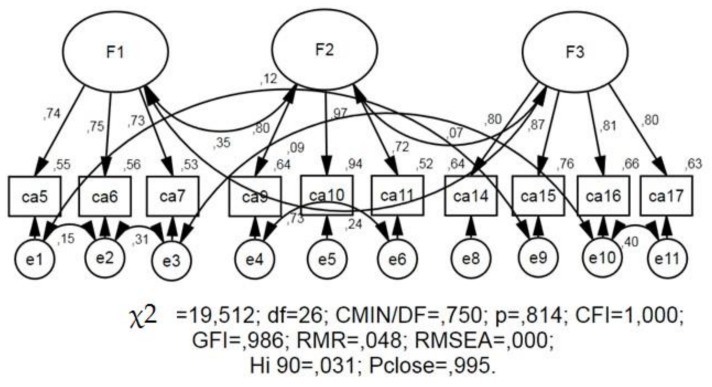
Confirmatory factor analysis for three-factor model of attitude toward cryptocurrency.

The confirmatory factor analysis performed by means of structural modeling in the SPSS Amos V.20 program confirmed the three-factor structure of the “Attitudes Toward Cryptocurrency Questionnaire” (Figure 1).

We found that all three subscales have high levels of internal consistency: Belief in the potential of cryptocurrency as a payment instrument (3, Cronbach’s alpha = 0.827), worries about the introduction of cryptocurrency in perspective (3, Cronbach’s alpha = 0.884), and willingness to use cryptocurrencies (4, Cronbach’s alpha = 0.900).

### 3.2. Representations of Bitcoin

At the pilot stage of the study, the task was to identify the semantic field of representations of Bitcoin. We were interested in the fact of how respondents perceive cryptocurrency: As money, as a new economic phenomenon (earning potential, improvement of economic status), or as a new technology. The choice of Bitcoin was explained by the fact that, compared with other types of cryptocurrency (more than 1900 types according to www.coinmarketcap.com), it prevails in the professional sphere, and specialists usually focus their attention on Bitcoin and less often on Ethereum and other types of cryptocurrency [13,33,34]. Bitcoin is the first and most famous cryptocurrency, with millions of transactions conducted. The popularity of Bitcoin is due to its characteristics: Decentralization (lack of regulation) and anonymity. Since our respondents are not experts in the field of cryptocurrency, we did not cover technically complex principles of its work and did not ask them about other, less popular cryptocurrencies. 

Results of association tests: Each respondent was asked to write several associations, arranging them in a certain sequence from one to three (one is the first association that arises in response to the presented stimulus, etc.). Bitcoin was presented as a stimulus word. The obtained data was processed using content analysis and subsequent frequency analysis. During the analysis, six categories of associations were identified: (1) Random associations, meaningfully unrelated to the concept of “Bitcoin” as a type of cryptocurrency (for example, “ball under water”, “porridge”, “game”, “meme”); (2) the symbol associated with the physical representation (associations that characterize the appearance: “Coin”, “code”, “image”, “password”); (3) associations expressing a negative attitude, doubt, or danger (“deception”, “bubble”, “risk”); (4) associations describing the prospects for using cryptocurrency and earning potential (“wealth”, “innovation”); (5) function (“payment”, “transaction”, “transfer”, “trading”); (6) associations associated with the evaluation of cryptocurrency properties as a global system (“Internet, ”technology”, “mining”).

The results of the frequency analysis showed that, first of all, young people associate Bitcoin with the possibility of earning and enrichment (26%), as well as with fears and doubts (23%). It was found that respondents who have experience in dealing with cryptocurrencies see its application as a potential source of income, a resource for improving their economic status. For this category of respondents, Bitcoin functions are attractive, i.e., easy to use (transaction, payment, transfer). They perceive Bitcoin as a sign of the current situation in the development of society—the growth of global processes (Internet, technology, mining). The group of associations which ranks second according to the respondents included judgments that have both positive and negative connotation modalities: 24% of respondents indicated “deception”, etc., and an equal percentage indicated earning potential. The third place was dominated by random associations (36%), which indicates the limited size of the semantic field of the phenomenon being studied. In addition, it can be assumed that the phenomenon of cryptocurrency still does not have clear boundaries in public discourse. This judgment agrees in many ways with the fact that at the stage of development and carrying out of the statistical procedure for checking the “Attitudes Toward Cryptocurrencies Questionnaire”, we have not statistically proven the value component.

### 3.3. Description and Factors of Attitude towards Cryptocurrency

The results of the main stage of the study were focused on solving two problems: (1) Identifying and analyzing the content of attitude to cryptocurrency components; (2) analysis of the socio-psychological factors of attitude to cryptocurrency components.

#### 3.3.1. Attitude Towards Cryptocurrency in the Group of Students

The results of the frequency analysis of the statements included in each of the three components of attitude toward cryptocurrency showed the following. In the cognitive component, the most important was the understanding of the inevitability of the development of technologies associated with electronic money (60.3% of respondents). At the same time, only a quarter of the survey participants (26%) were confident that within 10 years, cryptocurrency would be issued by the state and replace the money, and after 5 years, most of the stores in which they shop will accept payments in Bitcoin (28% of respondents). Thus, the time perspective of beliefs about the use of cryptocurrency is not presented to a high degree. While accepting the fact of the development of electronic money technologies, the majority of respondents disagreed that in the next 5–10 years, the cryptocurrency will become a familiar form of earning and enrichment, or receive the status of a means of payment. Frequency analysis of statements attributed to the affective component of the attitude revealed the ambivalence of experiences regarding cryptocurrency. Thus, in this group, the percentage of respondents who were anxious and worried about the fact that cryptocurrencies will be used by criminal groups for tax evasion and financial fraud and the percentage of young people who do not share these fears is about the same. It makes up about a third of the sample (34–35% each). At the same time, the percentage of respondents who are worried that “cryptocurrency is not secured except by the greed of people” (31%) is still slightly less than the percentage of those who are not worried about this (37%). The behavioral component of the relationship included three judgments pointing to passive forms of pre-submission of young people, and one to the willingness to use cryptocurrency. The results of the frequency analysis showed that the majority of young people who participated in the study do not search for information about the cost of cryptocurrency, and do not discuss it with friends and acquaintances (52%). They admit that they did not even catch themselves thinking about mining (52%). At the same time, the percentage of respondents who would buy cryptocurrency if they had the funds is slightly less (37%) than those who would not do this (39%).

In summary, we note that the attitude toward cryptocurrency in the group of young students, who took part in the study was formed to a greater degree as an understanding of the inevitability of the development of electronic money technologies and, to a lesser extent, it is expressed in the readiness for mining. Young people have not yet decided on whether or not to worry about the development of cryptocurrency. Some of them are frightened by the potential possibilities of its use by criminal structures. Thus, it is possible to make an intermediate conclusion: “Belief in the potential of cryptocurrency as a payment instrument” (subscale 1) are determined by the fact that it is inevitable; “Worries about the introduction of cryptocurrency in perspective” (subscale 2) are expressed in the form of fears about the criminalization of this sphere of financial transactions; “Willingness to use cryptocurrency” (subscale 3) is limited by the presence/absence of funds for its purchase. In general, the formation of the attitude toward cryptocurrency is mostly expressed in the cognitive component.

#### 3.3.2. Socio-Psychological Predictors of the Young Adults’ Attitudes Toward Cryptocurrency

The linear regression analysis conducted by using the method of reverse steps shows the following: Belief in the potential of cryptocurrency as a payment instrument (R = 0.611; R2 = 0.373; F = 21.629 with *p* < 0.001) is directly related to care for people (*β* = 0.738) and the value of freedom as independence in actions (*β* = 0.314), and is inversely related to purity (sanctity) and holiness (*β* = −0.488); it is positively associated with confidence in the national and global financial system (*β* = 0.120) and negatively associated with confidence in the government in the sphere of regulating the development of new technologies (*β* = −0.354). In addition, among the predictors, the genders (*β* = 0.223) and the ages of the respondents (*β* = 0.339) were identified.

Worries about the introduction of cryptocurrencies in perspective are directly related (R = 0.567; R2 = 0.321; F = 14.972 with *p* < 0.001) with a negative attitude to money and inability to change their financial situation (*β* = 0.239), focus on the value of self-confidence (*β* = 0.304), age (*β* = 0.434), confidence in national and global financial systems (*β* = 0.126), and confidence in governmental abilities to regulate the development of new technologies (*β* = 0.522). They are negatively related to attitude to people (*β* = −0.176) and to developers of cryptocurrency (*β* = −0.545), as well as to financial anxiety and distrust of others in money matters (*β* = −0.301). 

Finally, the willingness to use cryptocurrencies directly depends (R = 0.507; R2 = 0.257; F = 17.676 with *p* < 0.001) on respect for money and belief in its power (*β* = 0.387), orientation toward independence in actions (*β* = 0.319 ), gender (*β* = 0.120), and age (*β* = 0.172), and is also negatively related to confidence in the government in the sphere of regulating the development of new technologies (*β* = −0.246). 

## 4. Discussion

This article raised the problem of determination of youths’ attitudes to a new socio-economic phenomenon—cryptocurrency. Since the concept of “cryptocurrency” is poorly understood, we identified the semantic field of representations of Bitcoin, which showed that the main idea of the concept is the source of earnings. In the course of the research, a three-factor model of the attitude was confirmed, including: Beliefs in the potential of cryptocurrency as a payment instrument, worries about the introduction of cryptocurrencies in perspective, and willingness to use cryptocurrencies. Analysis of the contents of each component of the attitude to cryptocurrency in the group of young students showed that this phenomenon is perceived as inevitable due to the development of scientific and technological progress. Worries of young people about the criminalization of the sphere of financial transactions can apparently act as a stopper, inhibiting their willingness to use cryptocurrency as a way of earning.

Value orientations of the individuals, their moral foundations, their levels of confidence, and attitudes to money, as well as gender and age, were considered as socio-psychological factors of attitudes toward cryptocurrency.

Studies conducted within the framework of the theory of moral foundations indicate that the focus on caring for people belongs to liberal values, whereas the value of purity/sanctity and holiness is characteristic of the system of conservative beliefs [35]. In this regard, we can say that ideas about the prospects of cryptocurrency are primarily due to liberal views (values of care and independence, distrust of the government in the regulation of new technologies). Interestingly, ideas about the relationship of the future financial system with cryptocurrencies are supported by confidence in it. Apparently, the idea of the inevitability of the development of cryptocurrency relies on an understanding of the logic of the development of the financial system and its subjective predictability for respondents.

The close connection of distrust of cryptocurrencies with distrust of people and developers and, on the contrary, confidence in the government in regulating the development of new technologies, suggests that emotional distress and worries regarding the criminalization of cryptocurrency are primarily experienced by those who fear reducing control over the financial sphere and increasing the uncertainty of their own financial position. The direct connection of worries about introduction of cryptocurrencies with a negative attitude toward money and with the inability to change the financial situation is very indicative: It can be assumed that worries and anxiety regarding the use of new digital financial instruments by criminal structures arise primarily among those who build their economic well-being on external guarantors (state, employer) and not on themselves.

The data obtained confirm our assumption that those respondents who were willing to use cryptocurrency did so because of a desire for financial autonomy and distrust of social institutions. Indeed, the blockchain technology underlying cryptocurrencies reduces the need for centralized systems for regulating financial flows and ensures independence from interference in the transaction history. In addition, the results indicate that the high dynamics of the cost of cryptocurrency apparently stir up expectations of rapid enrichment in the presence of money orientation as a value, indicating trust in their power.

In summary, respondents who are characterized by liberal views and trust in the financial system tend to perceive cryptocurrencies as promising. Those respondents who are afraid of reducing control over the financial sphere and increasing uncertainty of their own financial position, shifting responsibility for their economic well-being to external guarantors rather than themselves, tend to experience worries about the introduction of cryptocurrency into everyday life. Finally, those young people who seek financial autonomy and do not trust social institutions are ready to use cryptocurrency. In general, economic activity, interest, and willingness to use cryptocurrencies, reflecting the desire for autonomy and financial independence, can be considered among indicators of successful economic socialization.

As a research perspective, it should be noted, firstly, that the questionnaire should be developed to increase the reliability of the value component of the attitude to cryptocurrency. Secondly, clarification of the role of attitudes towards cryptocurrency in the process of economic socialization of young people and identification of levels of economic socialization are needed.

## Figures and Tables

**Table 1:** Comparison of one-factor, two-factor, and three-factor models of attitude toward cryptocurrency.

	Chi Square	df	CMIN/DF; *p*	CFI	GFI	RMR	RMSEA	Pclose
One-factor model	96,81	41	2.361; *p* < 0.01	0.970	0.942	0.161	0.072	0.026
Two-factor model	62.758	42	1.494; *p* = 0.021	0.989	0.964	0.135	0.044	0.065
Three-factor model	19.512	26	0.750; *p* = 0.814	1.000	0.986	0.048	<0.001	0.995

## Availability of Data and Materials:

The datasets used and analyzed during the current study are available from the corresponding author upon reasonable request.
